# Validation and correlation of high-sensitive troponin I and troponin T in the emergency department

**DOI:** 10.1186/s12872-024-04230-1

**Published:** 2024-10-12

**Authors:** Moustafa. Nseir, Arash. Mokhtari, Mia. Stanisic, Ulf. Ekström, Ashkan Labaf

**Affiliations:** 1 Department of Cardiology, Lund University, Skåne University Hospital, Entrégatan 7, Lund, 221 85 Sweden; 2 Department of Internal and Emergency Medicine, Trelleborg Hospital, Trelleborg, Sweden; 3 Department of Clinical Chemistry, Lund University, Skåne University Hospital, Lund, Sweden; 4 Lund University, Department of Clinical Sciences, Lund, Sweden

**Keywords:** Troponin I, Troponin T, Correlation, Myocardial infarction, Prognosis, Sensitivity, Specificity

## Abstract

**Background:**

Troponin elevation is frequently observed in various scenarios in the Emergency Department (ED), yet there is a paucity of studies investigating simultaneously measured high-sensitivity cardiac troponin T (hs-cTnT) and troponin I (hs-cTnI) within a diverse cohort in a clinical setting.

**Methods:**

All patients who underwent troponin testing at a single center were eligible for this study. Only patients with simultaneous samples with hs-cTnI (Siemens) and hs-cTnT (Roche) were included, regardless of chief complaint.

**Results:**

Analysis of 1987 samples from 1134 patients showed a significant correlation between hs-cTnT and hs-cTnI (*r* = 0.86, *p* < 0.01). Of these samples, 65% exceeded the upper reference limit (URL) for hs-cTnT, and 30% for hs-cTnI with 39% who exhibited elevated hs-cTnT levels alongside normal hs-cTnI levels. The area under the curve (AUC) for acute myocardial infarction (AMI) for the index visit was 0.80 (95% CI; 0.75–0.85) for hs-cTnT and 0.87 (95% CI; 0.83–0.91) for hs-cTnI. Sensitivity and specificity were 91% and 39% for hs-cTnT, and 80% and 80% for hs-cTnI. Positive predictive value (PPV) and negative predictive value (NPV) was 9.3% and 98.5% for hs-cTnT respectively, corresponding for hs-cTnI was 21.3% and 98.3% respectively. Hazard ratios for 1-year mortality were 1.52 (95% CI; 1.40–1.66) for hs-cTnT and 1.26 (95% CI; 1.18–1.34) for hs-cTnI.

**Conclusion:**

Elevated troponins above the URL were very common in this diverse cohort, particularly for hs-cTnT, which was twice as frequent compared to hs-cTnI, resulting in low specificity and PPV for AMI.

**Supplementary Information:**

The online version contains supplementary material available at 10.1186/s12872-024-04230-1.

## Introduction

Diagnosing acute myocardial infarction (AMI) relies on the upper reference limit (URL) defined as the 99th percentile in a healthy population. There are various studies that have validated the diagnostic accuracy of high-sensitivity troponin T (hs-cTnT) and troponin I (hs-cTnI) in patients with chest pain for the diagnosis of AMI, with similar sensitivity and specificity [1, 2]. However, when employing the approved standardized clinical decision value based on the 99th percentile of healthy individuals, nearly one in five AMI patients have been identified as having inconsistent diagnoses when using hs-cTnI compared to hs-cTnT [3].

The hs-cTnI and hs-cTnT in the general population demonstrate only a moderate strength of correlation [4], but becomes notably stronger in the context of chest pain in the emergency department (ED) to rule out AMI, where the pretest probability is higher [5–7]. However, there is limited data available to provide a more comprehensive perspective within a more heterogenous clinical cohort, particularly in the context of lower pretest probability. Consequently, uncertainties remain regarding the correlation between the different isoforms in various contexts. Evidence suggests specific differences in biochemical properties, release dynamics, and association with specific comorbid conditions that might influence the clinical evaluation in the ED [8]. Thus, the lack of biological equivalence between the different hs-cTn assays should be emphasized, given the magnitude of hs-cTn samples analyzed in EDs every day.

Troponin testing in the ED without chest pain is frequent [9, 10], and interpreting results above the clinical decision values poses a challenge for clinicians. Additionally, there is the potential for downstream testing and unnecessary evaluations, which escalates expenses and imposes further burdens on the patient [9, 11]. Conversely, hs-cTn, particularly hs-cTnT, are correlated with increased mortality and morbidity, even in the absence of AMI. Thus, it has been suggested that elevated levels of hs-cTn in the ED could lead to increased attention towards these patients, even in the absence of chest pain, due to their elevated risk of morbidity and mortality.

The aim of this study was to investigate the correlation between hs-cTnI (Siemens) and hs-cTnT (Roche) in a diverse clinical cohort at the ED, as well as their association with AMI and mortality.

## Method

### Study design and population

This study used a prospective observational cohort study design carried out at single center from 1/12/2020 to 7/6/2022. All patients presenting to the ED at the hospital in Trelleborg, situated in the Skåne region of Sweden, who underwent troponin analysis at the physician’s discretion, were eligible to participate in the study. In addition, patients admitted to wards where hs-cTn samples were ordered were included. All laboratories in the region substituted their Cobas instruments from Roche for Atellica instruments from Siemens during a 2-year period which was one of the reasons to why the project was initiated. Blood samples were collected from patients who required troponin analysis and, hs-cTnI was analyzed at the local laboratory in Trelleborg on a Atellica analyzer and hs-cTnT analyzed at the laboratory in Malmö on a Cobas analyzer. The physicians had access to both troponin results in the electronic medical records (EMR).

The inclusion criteria were, therefore, that all patients at the ED and the Department of medicine who were investigated by a hs-cTn analysis were included in the study, irrespective of the presenting symptom or final diagnosis. Only subjects with simultaneous hs-cTnT and hs-cTnI analyzed, were included. Information about study participation was made available on the Trelleborg hospital’s website, allowing patients to take part of the information. Only patients aged 18 and older who did not express any objections to participation were included in the study. This study was approved by the Regional Ethics Review Board of Lund, EPN.

### Data collection and outcomes

Collection of the data was carried out through the data registry of the Clinical chemistry and Pharmacology department. The collected data included age, gender, test time, final diagnosis, presence of heart failure, reason for ED visit, creatinine and cTn levels. AMI diagnosis was evaluated and assessed by examining physician at the treating ward. Data extraction was performed using the backend system of the electronic medical records. Two cardiologists independently reviewed the medical records in selected cases where the hs-cTnT and hs-cTnI were not concordant. Participant deaths were identified until 07/06/2022 with the help of the Swedish National Cause of Death Register [12], from which the deceased date was extracted. Final diagnoses, based on ICD codes, were only collected if a discharge summary was available. The primary outcomes were the correlation between troponin levels in the samples, the sensitivity and specificity for AMI, and the association with 1-year mortality risk and kidney function in relation to both assays.

### Study definitions

Serial measurements were defined as having 24 h or less between each sample. This 24-hour timeframe was established to align with daily routine checks, with troponin samples, for admitted patients. Glomerular filtration rate (GFR) was estimated using the patients first registered creatinine level (taken in conjunction with the first blood sample to analyze hs-cTn, if clinically relevant) by using the CKD-EPI equation. The non-gender-specific 99th percentile for hs-cTnT (Roche) is 14 ng/L [13]. The 99th percentile for hs-cTnI (Siemens) is 34 ng/L in females, 53 ng/L in males and 45 ng/L in a non-gender-specific population [14, 15]. Elevated cTn were defined as values above the 99th percentile, while normal levels were defined as below that level.

### Statistical analysis

Statistical analysis was carried out by the IBM SPSS Statistic, version 27. Baseline characteristics are presented as mean ± SD or median and IQR for continuous variables and proportions for categorical variables. Only the first sample for hs-cTn is considered in baseline characteristics due to patients having different numbers of samples. Hs-cTnT and hs-cTnI were logarithmically transformed using a base 10 to achieve a more normal distribution and for parametric statistical tests between the variables. The statistical analyses were either sample-related or patient-specific. Pearson correlation coefficient (r) was used for linear correlation between hs-cTnT and hs-cTnI. To assess the sensitivity and specificity of hs-cTn to diagnose AMI, receiver-operating-characteristic (ROC) analysis was used. Only the first sample of each hs-cTn, at presentation, was used for the ROC analysis. Cox regression analysis was used to evaluate impact of hs-cTn on mortality risk. The analysis was based on the highest recorded levels of hs-cTn in patients, which were logarithmically transformed with the natural base (e). Survival time was determined from the date of the initial blood sample collection until the end of the study. Mann-Whitney U test was utilized to compare continuous variables. MedCalc statistical software [16] was used to determine sensitivity, specificity, negative predictive value (NPV) and positive predictive value (PPV), with 95% confidence intervals. All hypothesis testing was 2-tailed and statistical significance was defined as *p* < 0.05.

## Results

### Demographics

**Fig. 1:**
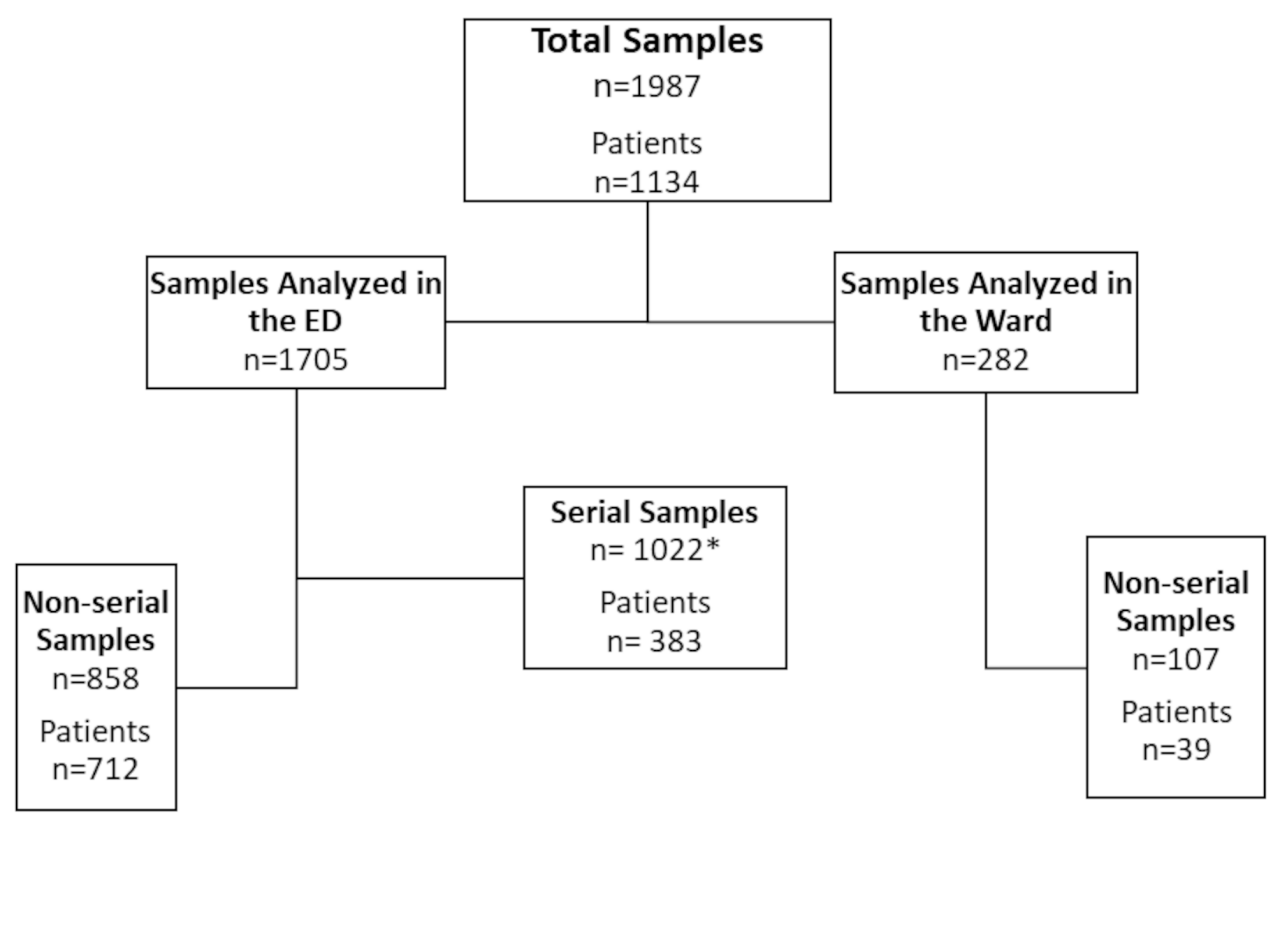
Flowchart visualizing the collection of blood samples. Serial samples are defined as having ≤ 24 h between the samples, while non-serial samples are defined as > 24 h between samples. Each sample refers to the measurement of a troponin pair consisting of hs-cTnT and hs-cTnI. * Refers to serial samples from ED and ward

Hs-cTn was analyzed in a total of 1134 patients, between 01/12/2020 and 27/08/2021. There were a total of 1987 blood samples where both hs-cTnI and hs-cTnT were measured (Fig. 1). There were 1022 serial and 965 non-serial samples. Baseline characteristics are presented for all patients in Table 1, distributed by the diagnosis of AMI. Hs-cTn was analyzed only once in 624 (55%) patients and twice or more in 510 (45%) patients, which included serial measurements (within 24 h) and/or different ED visits. Serial hs-cTn analysis took place in 424 (37%) patients out of the 510. The mean time between the serial blood samples was 3.7 ± 4.8 h.

**Table 1:** Baseline characteristics of patients

		All	AMI	No AMI
Number of patients (% of all)		1134	70 (6%)	1064 (94%)
Male		619 (54%)	36 (51%)	583 (55%)
Most common reason for ED visit	Chest pain	295 (26%)	35 (50%)	260 (24%)
	Dyspnoea	278 (25%)	12 (17%)	266 (25%)
	Arrhythmia	99 (9%)	1 (1%)	98 (9%)
	Stroke	44 (4%)	1 (1%)	43 (4%)
Heart failure		244 (22%)	19 (27%)	225 (21%)
Age, years		73 ± 14	74 ± 13	73 ± 14
Hs-cTnT (ng/L)		18 (10–34)	74 (28–244)	17 (10–31)
Hs-cTnI (ng/L)		14 (6–37)	242 (65-1608)	13 (6–30)
Creatinine (µmol/L)*		90 (74–114)	91 (71–115)	90 (74–114)
eGFR (mL/min/1.73m^2^)*		69 ± 24	68 ± 21	69 ± 24

Gender, reason for ED visit, heart failure and deaths are presented as percentage of all patients and of patients with and without AMI. Age, troponin concentrations, creatinine and eGFR are presented as mean ± SD or median (IQR) of the group. * Only 884 patients had creatinine levels measured

**Fig. 2:**
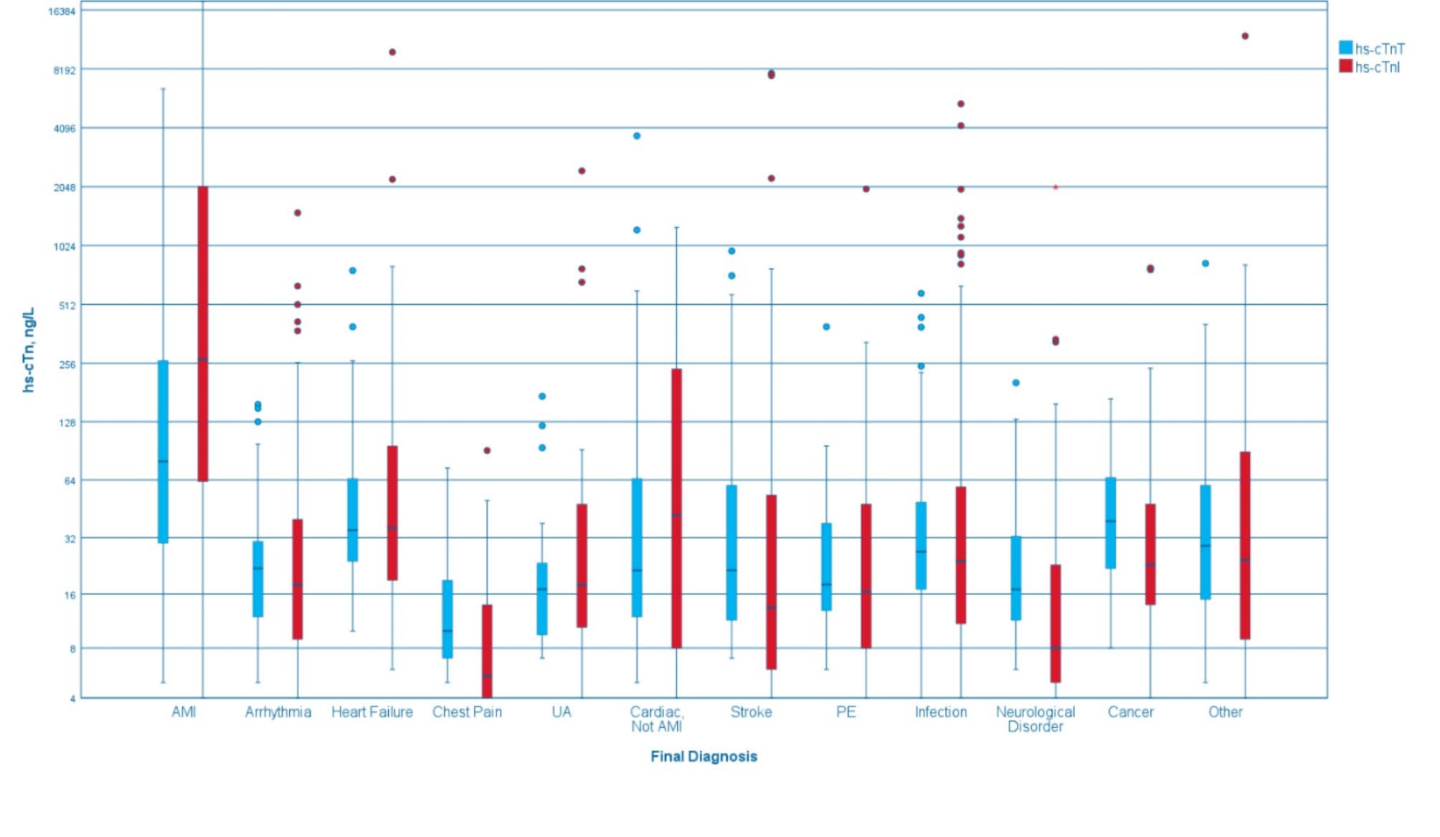
Boxplot showing the final diagnoses. Only unique final diagnoses per patient are displayed. A total of 1012 diagnoses are included. Red whiskers display hs-cTnI, while blue whiskers display hs-cTnT. Unstable angina is abbreviated as UA, while pulmonary embolism is abbreviated as PE. The y-axis represents hs-cTn levels in ng/L on a logarithmic scale for better visualization of the data range. Outliers are marked as individual points beyond the whiskers

There was a total of 1293 ED visits in which cTn measurement was carried out. Chest pain and dyspnea were the most common reasons for ED visit. Only 43 out of the 1134 included patients had no contact with the ED and had their hs-cTn levels measured solely within the ward. Final diagnoses are presented in Fig. 2.

### Troponin correlation

**Fig. 3:**
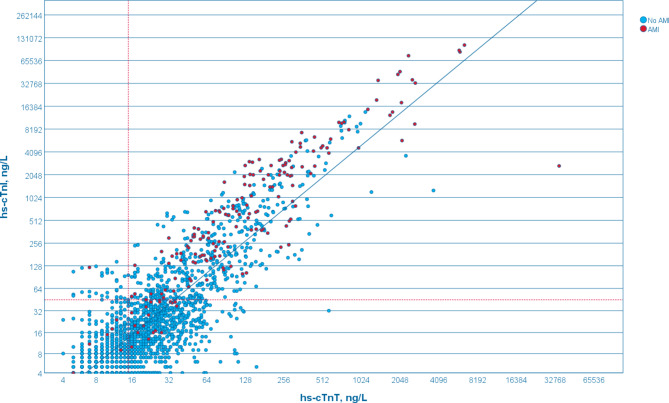
Scatterplot of hs-cTnT and hs-cTnI in all patients. All blood samples are included (1987). Each dot represents one blood sample. The Y- and X-axes are log2-scaled to enhance visibility. Non-gender-specific 99th percentile upper reference limit (URL) for both cTn are marked with dotted red lines. Regression line can be seen in blue. Red dots refer to AMI-diagnosed patients, while blue dots represent those without an AMI diagnosis

There was a significant positive correlation between hs-cTnT and hs-cTnI in males and females (Fig. 3), Pearson correlation, *r* = 0.86, with *p* < 0.001. Among the 1987 blood samples tested, 1287 (65%) showed elevated levels of hs-cTnT, whereas only 602 (30%) displayed elevated levels of hs-cTnI. Notably, 566 out of these 602 samples (92%) also demonstrated high levels of hs-cTnT.

Among the 1293 blood samples from ED, 37% exhibited levels below URL for both hs-cTnT and hs-cTnI, while 20% demonstrated levels above URL for both analyses. Additionally, 42% had levels above URL for hs-cTnT but below for hs-cTnI, while only 1% demonstrated the opposite pattern, with hs-cTnT levels below URL but hs-cTnI levels above it (Table 2).

**Table 2:** Categorization of blood samples by troponin levels and gender in emergency department visits

	All	Male	Female
Total visits	1293	691	602
TnT+/TnI+	256 (20%)	122 (18%)	134 (22%)
TnT-/TnI-	481 (37%)	236 (34%)	245 (41%)
TnT+/TnI-	538 (42%)	328 (47%)	210 (35%)
TnT-/TnI+	18 (1%)	5 (0.7%)	13 (2.2%)

Blood samples in patients admitted to the emergency department (ED) categorized based on hs-cTn above or below the 99th percentile of each assay. Only first blood sample is analyzed per ED visit. Levels above 99th percentile are marked as positive, while levels below 99th percentile are marked as negative

### Acute myocardial infarction

AMI was the final diagnosis in 6% of patients. Levels of hs-cTnT and hs-cTnI based on the final diagnosis are presented in Fig. 2. Almost all patients, 66 (94%), with AMI had high levels of both hs-cTnT and hs-cTnI. Three patients had elevated hs-cTnT but normal hs-cTnI levels, while one patient had elevated levels of hs-cTnI and normal levels of hs-cTnT (See Supplementary).

**Fig. 4:**
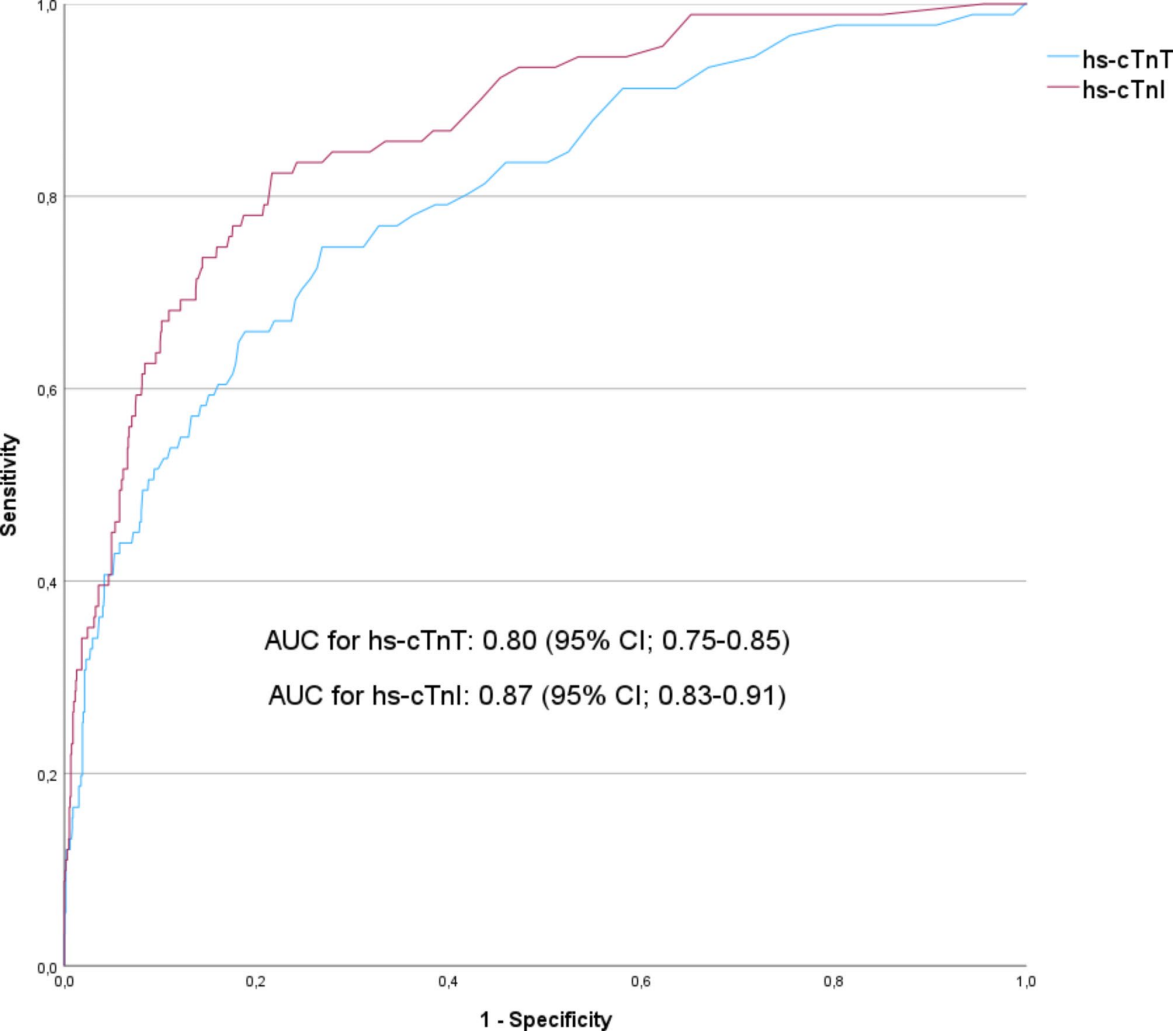
Receiver operating characteristic (ROC) curves show the diagnostic accuracy for AMI in hs-cTnT and hs-cTnI. Area under the curve (AUC) for both hs-cTn is also displayed. Red line represents hs-cTnI and blue line represents hs-cTnT

Diagnostic accuracy of AMI for the index visit was assessed through AUC, resulting in an AUC of 0.80 (95% CI; 0.75–0.85) for hs-cTnT, and 0.87 (95% CI; 0.83–0.91) for hs-cTnI (Fig. 4). Sensitivity for AMI in hs-cTnT was 91.2% (95% CI; 83.4–96.1), while specificity was found to be 39.2% (CI; 36.5–41.9). The NPV was 98.5% (95% CI; 97.1–99.2), and the PPV was 9.3% (95% CI; 8.7–10.0). For hs-cTnI, sensitivity was determined to be 80.2% (95% CI; 70.6–87.8), with specificity at 79.6% (95% CI; 77.3–81.8). The NPV was 98.3% (95% CI; 97.5–98.9) and PPV was 21.3% (95% CI; 18.9–23.9).

### Mortality risk

At the end of the study, a total of 173 patients had died, with 164 patients dying within one year of the first measured troponin value. Out of the 173 patients who died, only nine patients were diagnosed with AMI during the study. The Cox regression analysis showed a significant increase in 1-year mortality risk in patients with elevated hs-cTnT. A similar trend was observed for hs-cTnI. Hazard ratio (HR) was 1.52 (95% CI; 1.40–1.66, *p* < 0.001) for hs-cTnT, and 1.26 (95% CI; 1.18–1.34, *p* < 0.001) for hs-cTnI. Among the 173 patients who died, none exhibited normal levels of hs-cTnT, whereas 17 patients showed normal levels of hs-cTnI.

### Kidney function and troponins

**Fig. 5:**
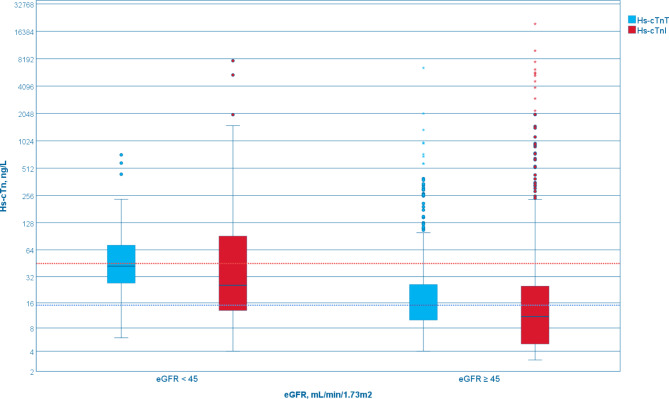
Boxplot demonstrating hs-cTn levels based on eGFR higher or lower than 45 mL/min/1.73m^2^. A significant difference in both hs-cTn was found between the groups (*p* < 0.001). Dotted blue line shows the 99th percentile upper reference limit (URL) for hs-cTnT (14 ng/L), while the dotted red line shows the non-gender specific 99th percentile URL for hs-cTnI (45 ng/L). Red whiskers display hs-cTnI, while blue whiskers display hs-cTnT

Among the 1134 patients, 884 individuals underwent measurement of their creatinine levels, enabling the calculation of the eGFR. The mean eGFR was 69 ± 24 mL/min/1.73m^2^, with median hs-cTnT being 18 (IQR, 10–34) ng/L, and 13 (IQR, 6–32) ng/L for hs-cTnI. Among these, 730 patients had an eGFR of 45 mL/min/1.73m^2^ or higher, while 154 patients had an eGFR lower than 45 mL/min/1.73m^2^. A significant difference in both hs-cTn (*p* < 0.001) was found between the two groups (Fig. 5), with lower eGFR resulting in higher levels of both hs-cTn.

Median hs-cTnT in patients with eGFR lower than 45 mL/min/1.73m^2^ was found to be 42 (IQR, 27–73), leading to 148 (96%) patients having hs-cTnT levels above URL. In contrast, median for hs-cTnI was found to be 26 (IQR, 13–93) in the same group, resulting in 55 (36%) patients having hs-cTnI levels above URL, using sex-specific cut-offs. Mean eGFR was found to be 31 ± 10 mL/min/1.73 m^2^. A weak negative correlation was observed between hs-cTnT and eGFR (Pearson correlation, *r* = -0.359, with *p* < 0.001). Additionally, a very weak negative correlation was found between hs-cTnI and eGFR (Pearson correlation, *r* = -0.180, *p* = 0.006). A total of six patients had AMI and eGFR lower than 45 mL/min/1.73m^2^.

## Discussion

In this prospective observational study encompassing patients with ED visits due to varying presenting complaints, we present three major findings.

First, a strong correlation was observed between the different hs-cTn assays. Previous studies have suggested a moderate correlation in the general population [4] and a strong correlation in individuals presenting with chest pain between the hs-cTnT and hs-cTnI [5–7], while our data exhibits a robust correlation in the ED within a more diverse cohort. Despite the strong correlation, there was however a notable discrepancy in the proportion of patients with elevated levels: 65% exhibited increased hs-cTnT levels, whereas only 30% had increased hs-cTnI levels with the majority of these patients not having an AMI. This discrepancy resulted in a low specificity and PPV for AMI, particularly for hs-cTnT in this diverse cohort. In 41% of samples there was a discrepancy between the assays, where the most substantial proportion being hs-cTnT+/hs-cTnI- with only 1% of the samples resulting in hs-cTnT-/hs-cTnI+. This is in line with a previous study where 8% and 40% had elevated levels of hs-cTnI and hs-cTnT, respectively [17]. There is a lack of mechanistic explanations for why hs-cTnT is released during various illnesses, while hs-cTnI appears to be more specific to cardiac myocyte injury. However, this phenomenon has been observed in various contexts [18, 19]. There also seems to be a discrepancy between the approved clinical decision value for hs-cTnI and its lack of biological equivalence to hs-cTnT [20]. Furthermore, the introduction of gender-specific cut-offs for hs-cTnI contributes to amplifying this disparity.

There was on the other hand 3 patients diagnosed with an AMI who only had elevated hs-cTnT resulting in a lower sensitivity for the hs-cTnI assay. The hs-cTnT levels were however only 16 ng/L in two of these patients who may have been misdiagnosed.

Secondly, an elevated hs-cTn-level was significantly associated with increased mortality risk. This association was more pronounced for hs-cTnT than for hs-cTnI, as previously shown [5, 21, 22]. Among the 173 deceased patients, none had a normal level of hs-cTnT and only 17 had a normal hs-cTnI level. Previous studies have shown similar results, with hs-cTnI seeming to have a closer association with composite CVD and coronary outcomes, whereas hs-cTnT was more strongly associated with the risk of all-cause mortality in various illnesses beyond AMI, leading to elevated values in patients with comorbidities [9]. In instances such as advanced kidney disease, atrial fibrillation, and chronic heart failure, hs-cTnT frequently demonstrates a more substantial increase and holds greater prognostic significance [23–27]. Although having a large proportion of ED having elevated hs-cTnT with only a minority having an AMI leads to a lower specificity and diagnostic challenges for the ED physician, this should still be seen as a negative prognostic marker.

Thirdly, among patients with an eGFR below 45, a substantial majority (96%) had hs-cTnT levels above the URL, compared to only one-third exhibiting elevated hs-cTnI levels. As noted in prior studies, all cTn assays exhibit slightly reduced sensitivity, and significant lower specificity in patients with CKD compared to those with normal kidney function. Moreover, optimal cut-off levels in CKD patients appear to be at least twice as high for Roche hs-cTnT and vary significantly between different assays [28]. Additionally, our findings demonstrate notably higher hs-cTn values compared to other studies involving CKD patients who present solely with chest pain, thus complicating direct comparisons. This indicates that the sensitivity, specificity, and optimal cut-off values in this heterogeneous cohort differ from those observed in studies involving solely patients presenting with chest pain.

There are a few limitations that need to be highlighted.

The presented data were obtained from a large cohort of heterogenous patients at a single urban hospital in Sweden which may affect the generalizability of our results to other ED settings. AUC for the troponin’s regarding AMI was not exclusive for patients with chest pain; We analyzed all patients that were admitted to the ED where troponin was sampled and ordered by the attending physician, regardless of the reason for the visit or the suspected diagnosis. This aspect is also a strength of the study. Not all patients had creatinine levels measured, which excluded a number of patients from our analysis due to lack of an eGFR. Most of the population had mildly reduced eGFR, making it challenging to compare groups in different CKD stages due to other group sizes being small. A large proportion of patients had only one cTn sample taken which decreased the available data for analyses of differences in troponin-dynamics. The reference assay in the presenting hospital was hs-cTnI, while hs-cTnT results were available in the EMR for all patients. Since hs-cTnT had been the assay used for many years previously and was familiar to the physicians, it could have influenced the AMI diagnosis, even though efforts were made to adjudicate inconsistent samples.

## Conclusion

Troponin elevation is very common among undifferentiated patients presenting to the ED. Although a robust correlation between hs-cTnT and hs-cTnI was found, elevated hs-cTnT levels were observed twice as frequently as hs-cTnI and were more commonly seen in those with a lower eGFR compared to hs-cTnI. This resulted in a lower specificity for hs-cTnT in the diagnosis of AMI. Those without AMI and with elevated hs-cTnT had a worse prognosis. Therefore, caution is advised when interpreting troponin levels in non-chest pain populations, particularly with hs-cTnT, due to its low specificity.

## Electronic supplementary material

Below is the link to the electronic supplementary material.

Supplementary Material 1

## Data Availability

The data used to support the findings of this study are available on request from the corresponding author.
