# Supplementary material for: Validation and correlation of high-sensitive troponin I and troponin T in the emergency department
Source: BMC Cardiovasc Disord. 2024 Oct 12;24:551. doi: 10.1186/s12872-024-04230-1 (PMC11470706; doi:10.1186/s12872-024-04230-1)
Supplement: Supplementary file 1 — Supplementary Material 1 [file 12872_2024_4230_MOESM1_ESM.docx]

Supplementary

Among the total patients diagnosed with acute myocardial infarction (AMI), four exhibited discordant results. Specifically, three patients had hs-cTnT levels above the URL but below hs-cTnI levels, while one patient demonstrated the opposite pattern. These patients were analyzed further by two independent cardiologists by reviewing medical records.

First patient, a female, presents to the ED with chest pain that began approximately a week ago. The patient is also presenting with infection symptoms. ECG at presentation shows a complete left bundle branch block, with hs-cTnT being 16 ng/L and hs-cTnI 10 ng/L. The patient is admitted to the ward where only hs-cTnI is assessed, consequently, their data is not incorporated into our database. Subsequently, during follow-up, hs-cTnI levels elevate to 171 ng/L. The patient is diagnosed with a minor non-ST elevation myocardial infarction (NSTEMI). Coronary angiography was also conducted without any signs of significant stenosis in the coronary arteries.

Second patient, a male, presents to the ED with chest pain where ECG indicates ST-elevation myocardial infarction. Both troponin markers are only analyzed at presentation, with hs-cTnT being 16 ng/L and hs-cTnI 30 ng/L. The patient is admitted to the ward where follow-up is exclusively monitored using hs-cTnT. Roughly two hours later, hs-cTnT levels increase from 16 ng/L to 90 ng/L. Percutaneous Coronary Intervention (PCI) is conducted the same day aimed at right coronary artery (RCA). The following day shows hs-cTnT value of 1656 ng/L.

Third patient, a male, presents to the ED with a history of chest pain triggered by exercise, accompanied by radiation to the jaw. The patient is assessed as acute coronary syndrome (ACS).

At presentation hs-cTnT was found to be 36 ng/L and hs-cTnI 42 ng/L, repeated troponin measurements showed no dynamic changes. The patient is admitted to the ward where further investigation shows left main coronary artery stenosis. A coronary artery bypass surgery was conducted.

Fourth patient, a female, presented with sudden chest pain and nausea lasting for 30 minutes while driving her car. The electrocardiogram (ECG) displayed no abnormalities. The hs-cTnI level was 119, while hs-cTnT was 7. Subsequent hs-cTnI samples indicated values of 122 and 114. An echocardiogram revealed no definite regional abnormalities and a normal ejection fraction of the left ventricle. Coronary angiography was performed, revealing no evidence of significant stenosis in the coronary arteries, although there was a tendency for spasm during the procedure. Additionally, an MRI was conducted, which did not reveal any signs of acute myocardial infarction (AMI), myocarditis, or fibrosis. The patient was diagnosed with NSTEMI, possibly attributed to spasm.

Hence, the causes of these discrepancies have been diverse. Firstly, establishing whether patients experienced AMI has proven challenging. Secondly, in some cases, patients had their hs-cTn sample collected in the ward, often referred to another center, utilizing only one of the troponin markers, resulting in the exclusion of these values from our database. Patient 1 and 2, had only simultaneous troponins at presentation with hs-cTnT just above URL at 16 ng/L, the subsequent dynamics in the following samples utilizing one of the assays. This makes the assessment of troponin disparity challenging.
